# Computed Tomographic Findings of Liver Cirrhosis in Dogs: Comparison between Compensated and Decompensated Cirrhosis

**DOI:** 10.3390/vetsci11090404

**Published:** 2024-09-01

**Authors:** Heesu Lee, Jaeeun Hyun, Kidong Eom, Jaehwan Kim

**Affiliations:** 1 Department of Veterinary Medical Imaging, College of Veterinary Medicine, Konkuk University, Seoul 05029, Republic of Korea; heesu002@konkuk.ac.kr (H.L.); eomkd@konkuk.ac.kr (K.E.); 2 Department of Veterinary Internal Medicine, College of Veterinary Medicine, Konkuk University, Seoul 05029, Republic of Korea; jaeeunhyun@konkuk.ac.kr

**Keywords:** cirrhosis, computed tomography, dog, hepatic

## Abstract

**Simple Summary:**

Cirrhosis is the end-stage of chronic hepatitis, characterized by the replacement of hepatic parenchyma with fibrous tissue and regenerative nodules. In cirrhotic livers, reduced hepatic function and interference with portal blood flow cause sinusoidal portal hypertension, resulting in secondary complications such as varices, ascites, and hepatic encephalopathy. In human medicine, computed tomography (CT) is the most sensitive diagnostic imaging technique used to evaluate hepatic morphological change. However, there is limited information on the CT features of cirrhosis in dogs. This study aimed to describe the CT characteristics of histologically confirmed cirrhosis and to compare these characteristics between compensated and decompensated cirrhosis. The results of this study can aid clinicians in diagnosing cirrhosis and predicting the progression of cirrhosis to the decompensated phase.

**Abstract:**

This study aimed to describe computed tomography (CT) characteristics of histologically confirmed cirrhosis and to compare these CT characteristics between compensated and decompensated cirrhosis. Sixteen dogs who underwent contrast CT and histopathological examinations were included; eleven dogs were assigned to the compensated group, and five dogs were assigned to the decompensated group. Irregular hepatic contours with a diffuse nodular distribution and hepatic lymph node enlargement are common concomitant features of cirrhotic livers on CT images. The enhancement patterns of the regenerative nodules and hepatic parenchyma were not significantly different from each other. Hypoattenuating areas with delayed contrast enhancement indicating fibrotic tissue were confirmed in 56.3% of cases. Hypoattenuating wedge-shaped area or nodule with minor or no contrast enhancement (histopathologically confirmed as focal necrosis) were confirmed in 37.5% of cases. Among CT variables, only normalized liver volume and portal vein-to-aorta ratio were significantly lower (*p* = 0.038 and 0.003, respectively) in the decompensated group. In conclusion, this study presented the CT features of cirrhosis and identified CT features that can discriminate between compensated and decompensated cirrhosis. Specifically, lower normalized liver volume and the portal vein-to-aorta ratio might be useful indicators for the progression of cirrhosis to the decompensated phase.

## 1. Introduction

Cirrhosis is the end-stage of chronic hepatitis and is characterized by fibrosis and regenerative nodules, resulting in the loss of normal liver structures [1,2,3]. In cirrhotic livers, reduced hepatic function and interference with portal blood flow cause sinusoidal portal hypertension [1,4]. This induces secondary complications, including varices, ascites, hepatic encephalopathy, hepatopulmonary hypertension, spontaneous bacterial peritonitis, coagulopathy, and severe jaundice [1,2,5,6,7,8]. Clinical signs are nonspecific and include anorexia, depression, vomiting, diarrhea, polydipsia/polyuria, melena, icterus, ascites, and hepatic encephalopathy, with ascites being the most common [1,2,8]. 

In humans, the development of ascites, gastrointestinal bleeding, hepatic encephalopathy, jaundice, and varices is defined as decompensated cirrhosis, which has a poorer prognosis than compensated cirrhosis (which has no or minor clinical signs) [9]. Recent studies in human medicine have reported that progressive atrophy of the whole liver occurs during the decompensated phase and that a significantly lower hepatic volume is associated with the development of decompensation [10]. Conflicting results regarding the prognosis of cirrhosis in dogs have been reported [1,2,11]; however, no reports have compared the clinical data and prognoses of compensated and decompensated cirrhosis in dogs.

Cirrhosis is diagnosed by integrating the clinical presentation and laboratory, imaging, and biopsy results. A definitive diagnosis can only be made through biopsy [4]; however, biopsies are associated with the risk of various complications, including bleeding, in the presence of hepatopathy [12]. Additionally, ultrasound-guided biopsy has limitations such as sampling error, semiquantitative, and subjective interpretation [13,14]. Various multimodal imaging features of cirrhosis have been studied in humans. Computed tomography (CT) is the most sensitive diagnostic imaging technique used to evaluate hepatic morphological changes [6,15,16]. However, little information is available regarding the CT features of cirrhosis in veterinary literature [17,18].

The purpose of this study was to characterize the qualitative and quantitative CT features in histologically confirmed cases of cirrhosis in dogs and to determine whether any specific imaging features can discriminate between compensated and decompensated cirrhosis.

## 2. Materials and Methods

### 2.1. Animals

Medical records of dogs that were presented to Konkuk University Veterinary Medical Teaching Hospital between 2019 and 2023 and had CT scans of the abdomen were evaluated for inclusion in the study using the terms “cirrhosis” and “chronic hepatitis”. The inclusion criteria were dogs that had a CT study of the abdomen and subsequent histopathologic diagnosis of cirrhosis. Histopathological diagnosis was performed through either incisional or tru-cut biopsy and referred to the IDEXX laboratory (Westbrook, ME, USA).

Medical records—including clinical signs, laboratory data, and histopathological reports—were retrospectively collected. Clinical and laboratory data (hematology, biochemistry, and coagulation panels) were recorded for each case at the time of the CT scans. As defined in human medicine [19], jaundice, ascites, varices, collaterals, and hepatic encephalopathy were confirmed through CT and clinical symptoms; patients were classified into the decompensated group. If only asymptomatic or nonspecific symptoms—such as decreased appetite, anorexia, and vomiting—were present, they were classified into the compensated group.

### 2.2. Computed Tomographic Examination

CT scans were conducted under general anesthesia in either sternal or dorsal recumbency using a 160-multislice CT (Aquilion Lightening 160; Canon Medical Systems, Otawara, Japan). Contrast medium (Omnipaque 300^®^; GE Healthcare, Chicago, IL, USA) with a dose of 2.5 or 3 mL/kg was injected via the cephalic vein at a 2–2.5 mL/sec infusion rate using a power injector (ADV CT 9000 injector; Liebel-Flarsheim, Cincinnati, OH, USA). The scanning parameters were 1–2.5 mm (slice thickness), 120–130 kVp (tube potential), and 80–180 mAs (current-time product). Single-phase CT scans included post-contrast images acquired 60–80 s after contrast medium injection. Three-phase CT scans included arterial-phase (AP), portal venous-phase (PP), and delayed-phase (DP) images. The AP image was acquired using the bolus tracking method, wherein the region of interest (ROI) was set at the descending aorta and scanned at a threshold of 200 HU. The PP and DP were imaged 20 s and 110–120 s after AP imaging, respectively.

### 2.3. Computed Tomographic Evaluation

CT images were saved as “Digital Imaging and Communication in Medicine” files and retrospectively reviewed using the postprocessing software RadiAnt (2024.1.0.34.2 version, Medixant, Poznan, Poland) for hepatic volumetry and INFINITT (Infinitt Health Care, Seoul, Republic of Korea) for other measurement and evaluation. For the three-phase images, pre-contrast, AP, PP, and DP images were evaluated. For single-phase images, pre- and post-contrast images were evaluated.

**Figure 1:**
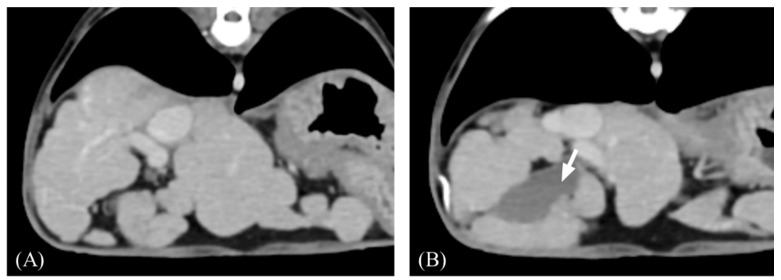
Representative transverse post-contrast computed tomography (CT) images of an irregular liver margin and gallbladder indentation. (**A**,**B**) Irregular margin showing nodular hepatic contour. (**B**) The gallbladder wall is sharply compressed by nodules (arrow). Slice thickness = 2.5 mm, Window = 400 HU, Level = 60 HU.

All measurements and evaluations were performed with a window width of 350 HU and a window level of 40 HU. All attenuation value measurements were conducted in each phase and measured in HU within the manually drawn circular ROIs. Each case was reviewed based on various qualitative and quantitative features. Qualitative CT features included the liver margin (smooth/irregular), nodule distribution (uniform/uneven), presence of gallbladder indentation, hepatic lymph node (LN) enlargement (>5.3 mm) [20], portal vein thrombosis (PVT), and transient hepatic attenuation difference (THAD). Liver margination was defined as irregular when any portion of the hepatic contour was distorted by nodules or capsular retraction (Figure 1). A uniform distribution was defined as the presence of nodules in all hepatic lobes. When nodules were identified in one or some of the hepatic lobes, the distribution was defined as uneven and the lobe in which the nodule was distributed was recorded. Gallbladder indentation was defined as a sharp compression of the gallbladder wall by the surrounding liver parenchyma or nodules (Figure 1B). THAD was recorded when early enhancement of the peripheral portal veins and a wedge-shaped hyperattenuating area were observed on the AP and became iso-dense to the liver parenchyma. Hepatic LN width was measured on the transverse DP image at the level of the porta hepatis. The presence of hypoattenuating areas on pre-contrast images, nonenhanced areas, or nodules was subjectively evaluated and recorded. Organ features other than ascites or varices indicating portal hypertension were also recorded.

**Figure 2:**
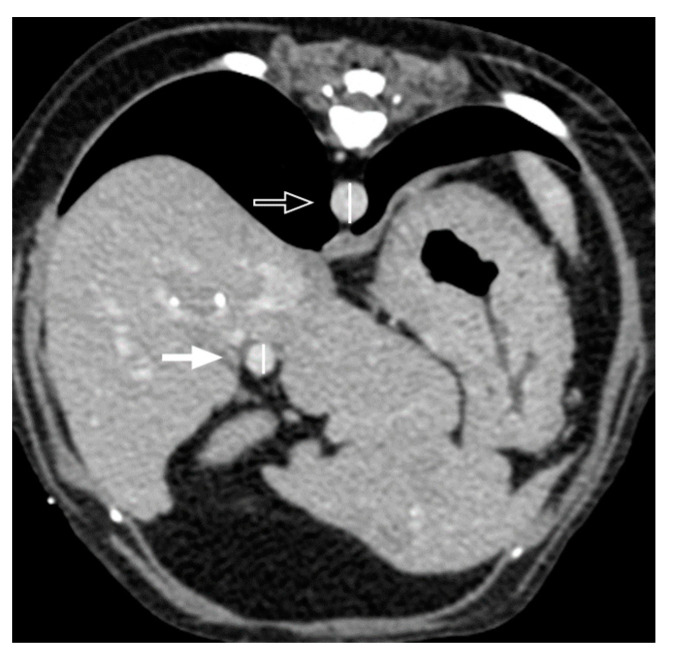
Transverse postcontrast CT images to measure the portal vein-to-aorta (PV/Ao) ratio. The portal vein (arrow) and aorta (open arrow) were measured at the level of porta hepatis. In this patient, the diameter of the portal vein and aorta measured at the level of portal hepatis is 6.5 mm and 8.4 mm, respectively, thus the ratio of the portal vein to the aorta is 0.77.

**Figure 3:**
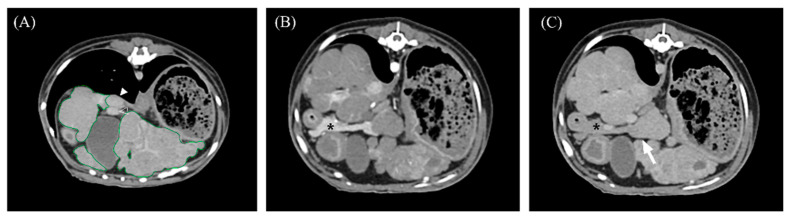
Portal venous phase (**A**,**C**) and arterial phase (**B**) transverse abdominal CT images for hepatic volume measurement. (**A**) The liver margin was manually drawn as a region of interest (ROI; green lines), including intrahepatic vasculature. The gallbladder, caudal vena cava (closed arrowhead), and extrahepatic portal vein (open arrowhead) were excluded from calculations. (**B**,**C**) The pancreas (asterisk) was clearly distinguished from the hepatic parenchyma in the AP, while the hepatic parenchyma and pancreas were indistinguishable in the PP (arrow).

Quantitative CT features included the attenuation value (HU) of the nodules and hepatic parenchyma, portal vein-to-aorta ratio (PV/Ao), and normalized hepatic volume (cm^3^/kg). The attenuation values of the nodules and hepatic parenchyma were measured at least five times in three different nodules to evaluate reproducibility. The mean value with standard deviation was used for evaluation and statistical analysis. If nodules were identified in the general hepatic parenchyma, ROIs > 15 cm^2^ were used to measure the attenuation value of the hepatic parenchyma. If nodules were focally identified in a specific lobe, they were compared with the hepatic parenchyma in the lobe where the nodules were not identified. The PV/Ao ratio was measured at the level of the porta hepatis on DP images (Figure 2). Hepatic volumetry was performed as described previously [21,22]. For single-phase scans, post-contrast images were used, while for three-phase scans, AP and PP images were used for further CT volumetric calculations. Over 20 cross-sections were manually drawn from the cranial margin of the liver at the diaphragm to the most caudal margin of the liver to measure the area of the hepatic parenchyma. Cross sections were selected as follows: 1, 1.25, 1.3, and 2.5 slice thicknesses drawn at two or three slice intervals for small dogs; 3.75, 3.9, or 4 slice intervals for medium-sized dogs; and 5 mm slice intervals for large dogs. Vessels in the hepatic parenchyma were included, whereas the extrahepatic portal vein, caudal vena cava, gall bladder, and extrahepatic bile duct were excluded. If the distinction between the pancreatic and liver parenchyma was unclear on the PP image, AP cross-sectional images were used (Figure 3). Hepatic volume was calculated using Equation (1) [21,22]:

Σ{each slice area (cm^2^) × slice thickness (cm)} × total number of slices of hepatic parenchyma ÷ number of slices used for calculation (1)

The hepatic volume was normalized by dividing the calculated hepatic volume by body weight. In the presence of ascites, body weight was measured after abdominal paracentesis.

Statistical analyses were performed using commercial statistical software (SPSS Statistics, Version 26.0; IBM Corp., Armonk, NY, USA; and Excel; Microsoft Corp., Redmond, WA, USA). All numerical variables are presented as means with standard deviations or medians with ranges. Fisher’s exact test was used to assess the qualitative CT data. The Kolmogorov-Smirnov test and Student’s t-test were used to assess the normality of numeric variables and compare the attenuation values of the nodules and hepatic parenchyma. Differences in hepatic volume, PV/Ao ratio, and laboratory results between the compensated and decompensated cirrhosis groups were assessed using the Mann-Whitney U test. Pearson’s correlations were used to identify correlations between the hepatic volume and PV/Ao ratio. For all comparisons, a *p*-value < 0.05 was considered statistically significant.

## 3. Results

### 3.1. Study Populations

Sixteen dogs met the inclusion criteria, including seven breeds: Maltese (*n* = 7), Cocker Spaniel (*n* = 3), Labrador Retriever (*n* = 2), Poodle (*n* = 1), Yorkshire Terrier (*n* = 1), Siberian Husky (*n* = 1), and mixed breed (*n* = 1). There were 11 castrated male dogs, one intact male dog, and four spayed female dogs. The mean age at diagnosis was 9.9 ± 3.3 (range: 1–15) years. Of the 16 dogs, 11 were referred for persisting hepatic enzyme elevation (*n* = 6), abdominal distension (*n* = 3), suspicion of hepatic tumor on abdominal ultrasound examination (*n* = 5), anorexia (*n* = 4), and jaundice (*n* = 1). The other five dogs initially presented with a skin mass (*n* = 2), urinary bladder calculi (*n* = 1), gingival mass (*n* = 1), and tracheal collapse (*n* = 1). Two dogs had bilateral, symmetrical skin lesions on the perianal and foot skin during physical examination at the time of admission, later diagnosed with superficial necrolytic dermatitis via skin biopsy.

Five dogs were classified in the decompensated group: ascites (*n* = 4), varices and collaterals (*n* = 4), and jaundice (*n* = 1). The patient with jaundice had esophageal varices and ascites and developed melena and hematemesis five months after diagnosis. One patient without peritoneal effusion presented with collateral vessels. All patients with peritoneal fluid underwent abdominal paracentesis and were confirmed to have transudates (*n* = 2) or modified transudates (*n* = 2). The compensated group included 11 dogs: 7 were asymptomatic and 3 had anorexia.

### 3.2. Qualitative Computed Tomographic Features

CT examinations included single-phase (*n* = 3) and three-phase (*n* = 13) scans. The qualitative CT features are summarized in Table 1. In all patients, liver margins were identified as irregular (100%). Nodules were uniformly distributed in 13 cases (13/16; 81.3%) and unevenly distributed in only three cases (3/16; 18.8%) in the compensated group. The nodules were unevenly distributed in one to three lobes, including the right lateral lobe, caudate process, papillary process, left medial lobe, and quadratic lobe. No commonly affected hepatic lobes were observed in the three unevenly distributed cases. In one case, nodules were only identified in the quadrate lobe; in the other two cases, only the right lateral lobe was affected. All patients in the decompensated group exhibited a uniform distribution (5/5, 100%). Gallbladder indentation was observed in seven cases (7/16; 43.8%), of which six were in the compensated group (6/11; 54.5%) and one was in the decompensated group (1/5; 20%). Peripheral LN enlargement was confirmed in 11 cases (11/16; 68.8%), of which eight were in the compensated group (8/11, 72.7%) and three were in the decompensated group (3/5; 60%). PVT was not observed in any patient. THAD was examined in 13 cases via triple-phase scan and was observed in seven cases (7/13; 53.8%), of which four were in the compensated group (4/8; 50%) and three were in the decompensated group (3/5; 60%). There were no significant differences in nodule distribution or the presence of gallbladder indentation, hepatic LN enlargement, or THAD between the two groups (*p* = 0.509, 0.308, 1.000, and 1.000, respectively). The qualitative CT features are compared in Table 1.

**Figure 4:**
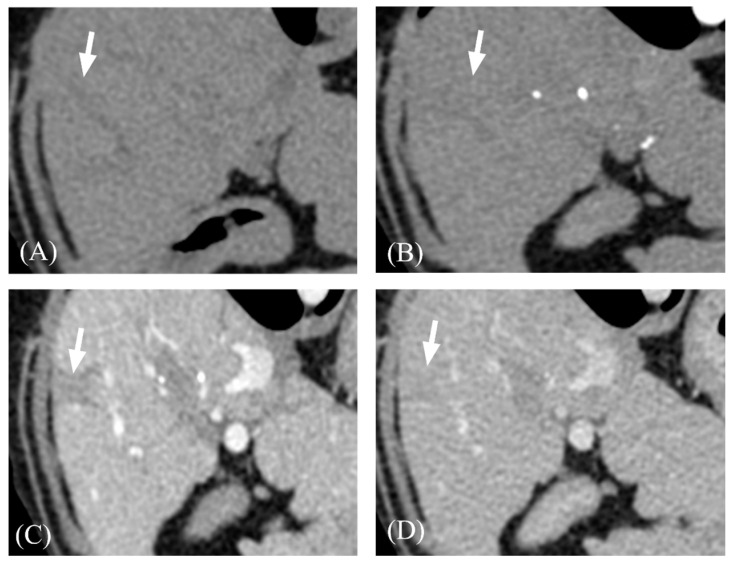
Representative three-phase CT images of focal hypoattenuating area with delayed contrast enhancement. (**A**) Pre-contrast. (**B**) AP. (**C**) PP. (**D**) DP. The hypoattenuating area with distinct margins (arrows) were gradually enhanced and showed its highest attenuation value at DP. Adjacent hepatic parenchyma showed its highest attenuation value at PP. Note that the lesion (arrow) became iso-attenuating to hepatic parenchyma on DP.

**Figure 5:**
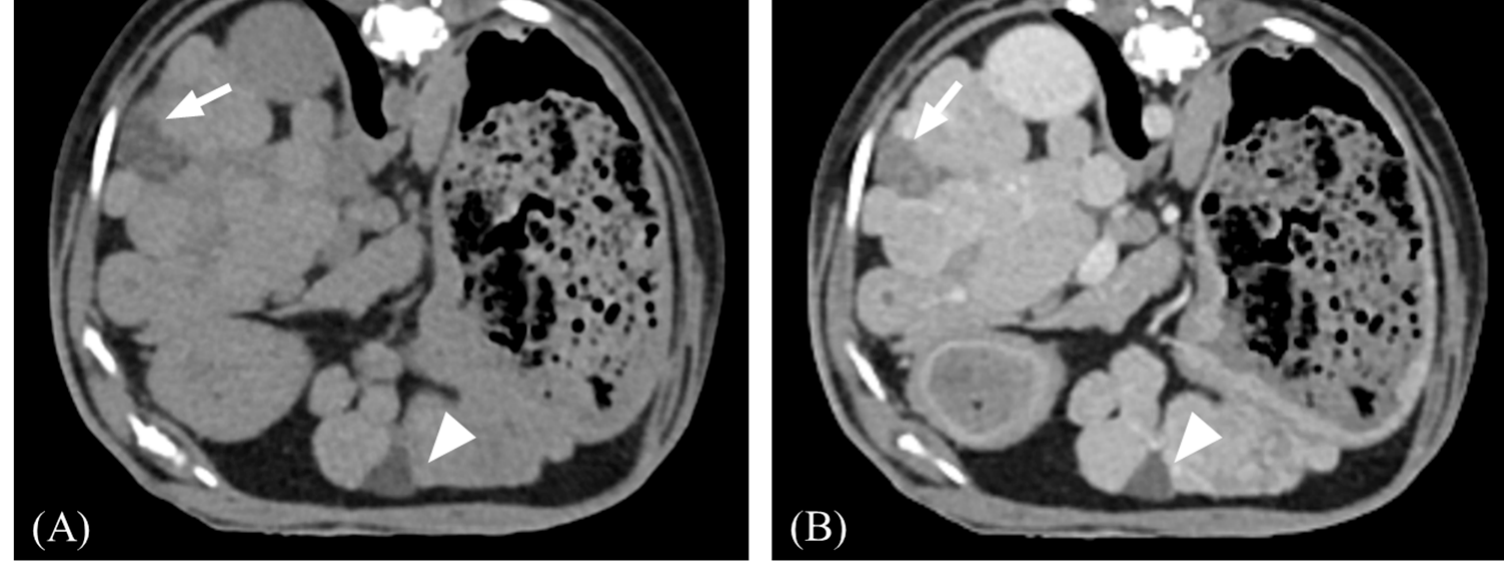
Representative CT images of a peripheral wedge-shaped hypoattenuating area. (**A**) Precontrast. (**B**) Postcontrast. Note that the wedge-shaped area (arrow) located peripherally shows a minor contrast enhancement. In contrast, the ventrally located lesion (arrowhead) was a cystic lesion, which showed fluid attenuation value with minimal enhancement.

Hypoattenuating areas on pre-contrast images showing delayed contrast enhancement were observed in nine cases (9/13; 69.2%; Figure 4). A wedge-shaped area or nodule showing hypoattenuation in all phases with minor or no contrast enhancement was identified in six cases (6/16; 37.5%; Figure 5).

**Figure 6:**
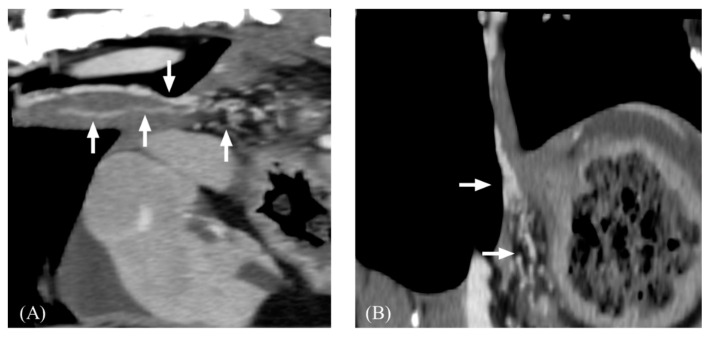
Post-contrast computed tomography images of esophageal varices. (**A**), sagittal plane. (**B**), dorsal plane. Note that tortuous small collateral veins (arrow) along the esophagus.

Varices and collaterals were confirmed in four of the five dogs in the decompensated group. Esophageal varices were only observed in two dogs (Figure 6); in the other two dogs, left gastro-azygos, splenorenal, porto-azygos, and splenorenal shunts were identified. In the case of porto-azygos and splenorenal shunts, pancreatic and gastric edema were observed. Mesenteric and gastric wall edema were also observed.

### 3.3. Quantitative Computed Tomographic Parameters

The quantitative CT features are summarized in Table 2. On each phase (pre-contrast, AP, PP, DP), the mean attenuation values of the nodules were 60.28 ± 5.75, 70.62 ± 21.78, 133.94 ± 34.8, and 126.14 ± 23.25 HU, respectively. Further, the mean attenuation values for hepatic parenchyma were 60.49 ± 6.61, 74.86 ± 29.96, 136.62 ± 29.68, and 128.82 ± 16.43 HU, respectively. There was no significant difference in the attenuation values between the nodules and hepatic parenchyma in each phase (*p* = 0.919, 0.739, 0.822, and 0.700, respectively; Table 2). The attenuation values for both the nodules and hepatic parenchyma were highest in the PP, and the margin of each nodule was most clearly visible in the PP.

Of the three cases with uneven nodule distribution, two were observed on three-phase scans and one was observed on a single-phase scan. The mean attenuation values of the nodules and hepatic parenchyma were 69.5, 58.7, and 72.6 HU, and 63.1, 64.2, and 59.5 HU on pre-contrast, respectively. In the AP, the mean attenuation values of the nodules and hepatic parenchyma were 116.2 and 70.7 HU, and 103 and 72.2 HU, respectively. On PP, the mean attenuation values of the nodules and hepatic parenchyma were 207.9 and 124 HU, and 161.6 and 122.6 HU, respectively. On DP, the mean attenuation values of the nodules and hepatic parenchyma were 190.5, 120.1, and 141.7 HU, and 164.2, 114.3, and 125.5 HU, respectively.

Normalized liver volume was significantly lower (*p* = 0.038) in the decompensated (26.5 ± 16.8) than compensated (47.6 ± 16.8) group. The PV/Ao ratio was significantly lower (*p* = 0.003) in the decompensated (0.93 ± 0.12) than compensated (1.19 ± 0.15; Table 3) group; however, there was no significant correlation between the normalized liver volume and PV/Ao ratio (r^2^ = 0.224, *p* = 0.405).

### 3.4. Laboratory Data

Laboratory data are summarized in Table 4. The median serum albumin concentration of the entire cohort was 2.9 (range: 2.0–3.5) g/dL (2.7 [range: 2.0–3.0] g/dL in the compensated group and 2.9 [range: 2.0–3.5] g/dL in the decompensated group). The median total bilirubin concentration of the entire cohort was 0.3 (range: 0.1–1.1) mg/dL (0.25 [range: 0.1–0.4] mg/dL in the compensated group and 0.6 [range: 0.2–1.1] mg/dL in the decompensated group). The median alkaline phosphatase (ALP) concentration of the entire cohort was 623 (range: 170–6536) U/L (483 [range: 198–6536] U/L in the compensated group and 2102 [range: 170–3134] U/L in the decompensated group). ALP concentrations were markedly elevated in all but two cases, of which one was in the compensated group, and one was in the decompensated group. The median aspartate transaminase (AST) concentration of the entire cohort was 80 (range: 38–287) U/L (70 [range: 38–89] U/L in the compensated group and 117 [range: 49–287] U/L in the decompensated group). In four cases, the AST level was within the normal range; three cases were in the compensated group, and one was in the decompensated group. The median alanine transaminase (ALT) concentration of the entire cohort was 317 (range: 56–793) U/L (260.5 [range: 56–431] U/L in the compensated group and 422 [range: 270–793] U/L in the decompensated group). In two cases, the ALT level was within the normal range; both were in the compensated group. The median gamma-glutamyl transferase (GGT) concentration of the entire cohort was 5 (range: 0–41) U/L (1.5 [range: 0–25] U/L in the compensated group and 34 [range: 5–41] U/L in the decompensated group). GGT level elevation was only observed in six dogs, of which four were in the compensated group and two were in the decompensated group. Albumin, total bilirubin, ALP, AST, ALT, and GGT concentrations were not significantly different between groups (*p* = 0.727, 0.373, 0.727, 0.376, 0.161, and 0.100, respectively).

## 4. Discussion

This study aimed to identify CT features in canine cirrhosis based on those established in humans. As the histopathological findings of cirrhotic livers are similar in humans and dogs, a similar finding was observed in the current study. Common CT findings of cirrhosis in this study included irregular hepatic contours with uniformly distributed nodules, hepatic LN enlargement, and the presence of a hypoattenuating area on pre-contrast images with delayed contrast enhancement, similar to those reported in humans [6,16].

Since cirrhosis is characterized by regenerative nodules and fibrosis [1,3], CT findings indicative of regenerative nodules and fibrosis strongly suggest a cirrhotic liver. In humans, regenerative nodules mainly receive blood flow from the portal vein and perform normal hepatocellular functions; additionally, they show contrast enhancement similar to that of the surrounding liver parenchyma [23]. By contrast, fibrotic tissue shows delayed, persistent contrast enhancement due to retention of the contrast medium [6,24].

Numerous regenerative nodules distorted the hepatic margin. As the fibrotic septa surrounding the regenerative nodules were hypoattenuating in the PP, the margins of the regenerative nodules were most prominent in the PP; in the DP, the margins were indistinguishable. Hypoattenuating areas with delayed contrast enhancement of variable thickness, which is consistent with bridging fibrosis in human medicine, were observed in 56.3% of cases. Conversely, our study did not identify CT findings indicative of focal confluent fibrosis, a major CT finding in human cirrhosis that is defined as a peripheral, wedge-shaped, hypoattenuating lesion causing capsular retraction [6,25]. There have been no reports on the presence of focal confluent fibrosis in dogs; thus, further histopathological studies with a larger number of cases are required to determine whether focal confluent fibrosis occurs in dogs.

Hypoattenuating areas and nodules, mostly located peripherally with distinct margins, were identified in 37.5% of cases. Histopathological examination revealed this to be focal necrosis in one case. Other cases may also be related to the necrotic region, consistent with previous studies wherein a measurement < 37 HU was theorized to represent a region of necrosis [26]. Additionally, previous studies in humans have reported that decreased portal blood flow induces necrotic, infarcted regenerative nodules and focal ischemic necrosis of the liver, presenting as hypovascular hypoattenuating lesions on CT [27,28,29,30,31]. Similarly, in dogs, as regenerative nodules become larger, the vascular supply is compromised, causing ischemia [8]. In humans, infarcted regenerative nodules are associated with variceal bleeding and liver hypoperfusion [28,29]. However, in the current study, no patients with hypoattenuating nodules had variceal or gastrointestinal bleeding. Considering the low prevalence of varices in the present study, further studies are required to determine whether variceal bleeding or other pathological conditions are associated with focal liver necrosis in dogs.

In all cases except for three dogs, the nodules were evenly distributed across all hepatic lobes. The three dogs exhibiting unevenly distributed nodules all belonged to the compensated group and were asymptomatic at the time of diagnosis; however, the ALP level was elevated more than three times the upper limit of the reference interval. This finding aligns with the results of a prior study wherein the histological diagnosis of cirrhosis was made in less than two out of six lobes in 42.9% of dogs who did not manifest clinical symptoms of liver disease [13]. Previous studies regarding histological variations in hepatopathies recommended biopsy of at least two liver lobes for an exact diagnosis [13,32]; however, performing multiple biopsies may increase the risk of complications [12]. In the current study, the incisional biopsy site was determined based on CT. Therefore, identifying the most likely lesion by using CT before performing a biopsy should be considered.

The correlation between hepatic fibrosis stage and stiffness of the hepatic parenchyma has been reported in both humans and dogs [4,33]. In our cases, it is likely that increased stiffness of the hepatic parenchyma compresses the adjacent gallbladder wall, causing a sharp indentation in the cross-sectional image. However, despite the decompensated group being in a more advanced stage of cirrhosis, only 20% of dogs in this group exhibited gallbladder indentation. This might be attributed to the presence of ascites between the hepatic lobe and gallbladder, as the remaining 80% of dogs in this group had ascites.

Hepatic LNs were enlarged in 68.8% of dogs, including two of three cases with uneven nodule distribution. In humans, lymphadenopathy in chronic liver disease has been reported in approximately 38–50% of dogs, with the frequency of enlarged LNs varying based on the etiology [34,35]. The higher incidence of enlarged LNs in dogs than in humans may be attributed to differing etiologies, with chronic hepatitis being the major cause of cirrhosis in dogs.

THAD can arise from various etiologies, including hepatic tumors, inflammation, cirrhosis, or even conditions unrelated to liver disease [36,37]. In cirrhosis, fibrosis-induced structural distortion of the microvascular anatomy impedes portal flow, leading to the formation of arterioportal shunts and resulting in THAD [38,39]. In the present study, THAD was identified in 53.8% of cases, which is much higher than in humans [38,40,41]. Previous studies in human medicine have reported that while arterioportal shunting occurs mainly via the trans-sinusoidal route, anastomoses, and changes in the permeability and capillarization of sinusoids also have an effect [38,39]. Similarly, in dogs, the vascular blood supply is compromised as regenerative nodules expand, causing the development of bridging vascular channels between the hepatic artery and portal vein [8]. It seems that the different mechanisms of arterioportal communication or other causes other than cirrhosis can affect the differences in prevalence between humans and dogs.

PVT is reported as a major complication of chronic hepatitis in human medicine, with a prevalence up to 20% that increases with disease severity [42,43]. However, a recent study reported a prevalence of 2.2% in dogs with chronic hepatitis, which is much lower than that in humans [44]. Similarly, in the present study, PVT was not observed in any patient, confirming that the occurrence of PVT secondary to cirrhosis is rare in dogs.

In human medicine, esophageal varices (EVs) develop in 30–70% of individuals with cirrhosis; hemorrhage from EVs stands as the primary cause of mortality with cirrhosis [45,46]. A previous study detailing EVs in dogs concluded that EVs are uncommon and likely to have minimal clinical importance compared with humans [47]. Interestingly, only two dogs in our study had EVs, one of whom developed hematemesis 5 months after the initial diagnosis. The remaining patient did not exhibit complications secondary to the EV.

Approximately one-third of dogs were classified into the decompensated group. In one case, only the collateral vessels were identified, with no evidence of ascites. In humans, the development of ascites is considered the final consequence of anatomical and pathophysiological abnormalities in dogs with cirrhosis [6]. Therefore, CT imaging may be helpful for identifying other complications, such as varices and collaterals, and for evaluating hepatic volume in the absence of ascites. Only the normalized hepatic volume and PV/Ao ratio were significantly lower in the decompensated group; other qualitative CT features were not significantly different between the groups. Liver size decreases with cirrhosis [1,2,8]; however, no studies in veterinary medicine have divided liver cirrhosis into compensated and decompensated stages to investigate liver size. In human medicine, more than 60% of patients with early cirrhosis exhibit hepatomegaly and progressive liver atrophy, and lower hepatic volume is reported during the decompensated phase [6,10]. This study also confirmed that dogs in the decompensated group had significantly smaller liver volumes. This study also found that three-phase imaging is beneficial for liver volume measurements, particularly due to the clear margination of the pancreas adjacent to the liver during the AP. Portal vein diameter increases as cirrhosis progresses and then decreases with hepatofugal or reversed flow, slower flow, and collateral shunt in humans [48,49]. Similarly, the PV/Ao ratio was significantly lower in the decompensated group, who were at a more advanced stage than the compensated group, suggesting that a similar mechanism may apply to dogs.

A recent study in humans reported that hepatic volume is inversely proportional to albumin levels [10]. However, there were no significant differences in laboratory results, including albumin, total bilirubin, ALP, AST, ALT, and GGT, between the compensated and decompensated groups in this study. Interestingly, contrasting with previous studies [2,8], all dogs except two showed normal albumin levels. The other two dogs, each in the compensated and decompensated group, respectively, exhibited mild hypoalbuminemia. It has been demonstrated that there are no significant associations between the degree of serum liver enzyme elevation, hyperbilirubinemia, serum albumin, and overall survival [2,11]. Thus, laboratory results alone cannot differentiate between the compensated and decompensated groups in dogs, raising the need for CT examination of cirrhotic dogs.

In this study, Maltese was the most affected breed (43.8%), followed by Cocker Spaniel (18.8%) and Labrador Retriever (12.5%). Cocker Spaniels and Labrador Retrievers have been reported to be predisposed to cirrhosis, with several genetic factors involved [1,2,11,50]. However, Maltese dogs have not been reported to be predisposed to cirrhosis. Considering the different breed distributions in each country, regional factors may have affected prevalence; thus, further studies are needed to determine whether genetic factors are involved in the development of cirrhosis in Maltese dogs.

This study has some limitations, including its retrospective design and small sample size. The small sample size prevented us from conducting meaningful statistical analyses beyond the included tests, including establishing cutoff values; thus, numerous cases are required to verify the aforementioned findings. Owing to the retrospective nature of the study, CT scans were not conducted under uniform conditions, which may affect the CT evaluation of each case. Furthermore, since CT scans were not conducted at the same stages of the disease course, there can be variations in CT findings depending on the stage of the disease. Additionally, all laboratory results were evaluated based on the initial presentation; however, outcomes may differ depending on disease progression. Finally, as this study only includes cirrhosis, other hepatic diseases can present similar CT findings. Further studies, including various hepatic diseases, should be conducted to confirm the pathognomonic findings of cirrhotic liver.

## 5. Conclusions

In conclusion, this study presented common CT features of cirrhotic liver in dogs. Therefore, the qualitative features and the quantitative parameters of CT examination can aid in the diagnosis of cirrhosis and discriminating between compensated and decompensated cirrhosis. Specifically, both lower normalized liver volume and portal vein-to-aorta ratio might be useful indicators for the progression of cirrhosis to the decompensated phase.

## Figures and Tables

**Table 1:** Evaluation of qualitative computed tomographic features.

		Total	Compensated	Decompensated	*p*-Value
Liver margination	Smooth	0/16 (0%)	0/11 (0%)	0/5 (0%)	
	Irregular	16/16 (100%)	11/11 (100%)	5/5 (100%)	
Nodule distribution	Uniform	13/16 (81.3%)	8/11 (72.7%)	5/5 (100%)	0.509
	Uneven	3/16 (18.8%)	3/11 (27.3%)	0/5 (0%)	
Gallbladder indentation	Present	7/16 (43.8%)	6/11(54.5%)	1/5 (20%)	0.308
	Absent	9/16 (56.3%)	5/11 (45.5%)	4/5 (80%)	
Periportal lymph node enlargement (>5 mm)	Present	11/16 (68.8%)	8/11(72.7%)	3/5 (60%)	1.000
	Absent	5/16 (31.3%)	3/11 (27.3%)	2/5 (40%)	
Portal vein thrombosis	Present	0/16 (0%)	0/11 (0%)	0/5 (0%)	
	Absent	16/16 (100%)	11/11 (100%)	5/5 (100%)	
Transient hepatic attenuation difference	Present	7/13 (53.8%)	4/8 (50%)	3/5 (60%)	1.000
	Absent	6/13 (46.2%)	4/8 (50%)	2/5 (40%)	

**Table 2:** Comparison of mean attenuation value of regenerative nodule and hepatic parenchyma on each phase.

		Attenuation Value (HU)		
	*n*	Regenerative Nodule	Hepatic Parenchyma	*p*-Value
Pre-contrast	16	60.28 ± 5.75	60.49 ± 6.61	0.919
Arterial phase	13	70.62 ± 21.78	74.86 ± 29.96	0.739
Portal venous phase	13	133.94 ± 34.8	136.62 ± 29.68	0.822
Delayed phase	16	126.14 ± 23.25	128.82 ± 16.43	0.700

Note: Data are presented as the mean ± standard deviation; *p* < 0.05 is considered significant; HU, Hounsfield unit.

**Table 3:** Computed tomography image variables of the compensated and decompensated groups.

	Total (*n* = 16)	Compensated (*n* = 11)	Decompensated (*n* = 5)	*p*-Value
Liver volume/body weight (cm^3^/kg)	41.0 ± 19.2	47.6 ± 16.8	26.5 ± 16.8	0.038 *
PV/Ao ratio	1.11 ± 0.18	1.19 ± 0.15	0.93 ± 0.12	0.003 *

Note. Data are presented as the mean ± standard deviation; *p* < 0.05 is considered significant. * *p* < 0.05; HU, Hounsfield unit; PV/Ao, portal vein-to-aorta thickness.

**Table 4:** Laboratory results in the entire case and with each group.

	Total (*n* = 16)	Compensated (*n* = 11)	Decompensated (*n* = 5)	*p*-Value
Albumin (g/dL)	2.9 (2.0–3.5)	2.7 (2.0–3.0)	2.9 (2.0–3.5)	0.727
Total bilirubin (mg/dL)	0.3 (0.1–1.1)	0.25 (0.1–0.4)	0.6 (0.2–1.1)	0.373
ALP (U/L)	623 (170–6536)	483 (198–6536)	2102 (170–3134)	0.727
AST (U/L)	80 (38–287)	70 (38–89)	117 (49–287)	0.376
ALT (U/L)	317 (56–793)	260.5 (56–431)	422 (270–793)	0.161
GGT (U/L)	5 (0–41)	1.5 (0–25)	34 (5–41)	0.100

Note: Data are presented as the median (range); ALP, alkaline phosphatase; AST, aspartate aminotransferase; ALT, alanine aminotransferase; and GGT, gamma-glutamyl transpeptidase.

## Data Availability

The data that support the findings of this study are available from the corresponding author upon reasonable request.
